# Prevalence, comorbidities, and factors associated with prolonged grief disorder, posttraumatic stress disorder and complex posttraumatic stress disorder in refugees: a systematic review

**DOI:** 10.1186/s13031-024-00586-5

**Published:** 2024-04-16

**Authors:** Franziska Lechner-Meichsner, Hannah Comtesse, Marie Olk

**Affiliations:** 1 Department of Psychology, Clinical Psychology and Psychotherapy, Goethe University Frankfurt, Varrentrappstraße 40-42, 60486 Frankfurt am Main, Germany; 2 Clinical Psychology, Utrecht University, Heidelberglaan 1, Utrecht, 3584 CS Netherlands; 3 Clinical and Biological Psychology, Catholic University Eichstaett-Ingolstadt, Ostenstraße 26, 85072 Eichstätt, Germany

**Keywords:** Refugees, Trauma, Prolonged grief disorder, Posttraumatic stress disorder, Complex posttraumatic stress disorder, Systematic review

## Abstract

**Background:**

The number of refugees worldwide is at an all-time high with many being exposed to potentially traumatic events and the loss of loved ones. The 11^th^ revision of the International Statistical Classification of Diseases and Related Health Problems now includes prolonged grief disorder and complex posttraumatic stress disorder and revised criteria for posttraumatic stress disorder. An overview of these stress-related disorders among people who have become refugees is therefore needed. Consequently, we conducted a systematic review to determine prevalence rates, comorbidities, and associated factors for each of the disorders.

**Method:**

We systematically searched PubMed, Web of Science, and PsycArticles to identify studies that reported prevalence rates, predictors or associated factors, and/or comorbid mental disorders for either (1) prolonged grief disorder, (2) posttraumatic stress disorder, or (3) complex posttraumatic stress disorder among refugees. The selection process followed the PRISMA guidelines.

**Results:**

A total of 36 studies met the inclusion criteria. Most of the studies were of high quality. There was substantial variation in prevalence rates by disorder, with prolonged grief ranging from 6 to 54%, posttraumatic stress disorder ranging from 0.4 to 80%, and complex posttraumatic stress disorder ranging from 3 to 74.6%. Pooled prevalence for posttraumatic stress disorder was estimated at 29.8% in treatment seeking samples and 9.92% in population samples. For complex posttraumatic stress disorder, it was estimated at 57.4% in treatment seeking samples and 7.8% in population samples. Posttraumatic stress disorder was among the most frequent comorbidities for prolonged grief disorder while depressive symptoms were the most frequently occurring co-morbidity across all three disorders. Sociodemographic variables, trauma exposure, and loss characteristics were associated with higher symptom severity. Postmigration living difficulties played an important role in prolonged grief and complex posttraumatic stress disorder.

**Conclusion:**

The review revealed substantial differences in prevalence rates between the three studied disorders but underscored a very high prevalence of ICD-11 stress-related disorders among refugees. The identified associated factors point to subgroups that may be particularly at risk and establishes a foundational basis for targeted interventions and potential policy changes. Future research should incorporate longitudinal investigations and emphasize culturally sensitive assessments.

**Supplementary Information:**

The online version contains supplementary material available at 10.1186/s13031-024-00586-5.

## Introduction

 The number of forcibly displaced people worldwide is at an all-time high; as of 2022, 104.8 million people were displaced, of whom 35.3 million were refugees [1]. In a survey of refugees recently arrived in Germany, 85.5% reported exposure to one or more potentially traumatic events (PTE), with 4.78 different PTE on average [2]. Interpersonal PTE, like experiencing physical violence and being exposed to death or murder, as well as suffering deprivation are most frequently reported among refugees [3].

Given the frequency and nature of PTE, it is not surprising that posttraumatic stress disorder (PTSD) is among the most frequent mental disorders in refugees, and has received considerable attention [4–6]. In the 11^th^ revision of the International Statistical Classification of Diseases and Related Health Problems (ICD-11; [7]) the criteria for PTSD were recently revised and the new diagnosis of complex PTSD (cPTSD) has been introduced in the section on disorders specifically associated with stress. ICD-11 was developed with a focus on clinical utility and international applicability [8] and PTSD symptoms that overlap with other mental disorders (e.g. sleep problems) were removed [9]. The ICD-11 criteria for PTSD (PTSD_ICD-11_) now focus on the core symptom clusters of (1) re-experiencing of the event as vivid intrusive memories, flashbacks, or nightmares; (2) avoidance of reminders that trigger thoughts and memories of the event; and (3) persistent sense of current threat [7]. ICD-11 criteria for PTSD are much narrower than previous criteria in ICD-10, and in the Diagnostic and Statistical Manual of Mental Disorders (DSM-5) where PTSD is described by 20 symptoms in four clusters [10]. Compared to DSM-5 criteria, ICD-11 criteria come with a higher diagnostic threshold [11] and less heterogeneity in the disorder [12].

The new sibling diagnosis of cPTSD is defined as a reaction to chronic or repeated traumatic events from which escape is difficult or impossible (e.g., repeated childhood abuse, prolonged domestic violence, torture, slavery). A diagnosis of cPTSD can be made when all PTSD_ICD-11_ criteria are fulfilled and disturbances in self-organization (DSO) are present. DSO consist of three symptom clusters: (1) affect dysregulation; (2) negative self-concept that includes feeling “diminished, defeated, or worthless”, and is accompanied by feelings of shame, guilt, or failure; and (3) difficulties in relationships [7]. The diagnosis of cPTSD replaces enduring personality change after catastrophic experience (EPCACE) in ICD-10 and does not exist in DSM-5.

To date, no systematic review on PTSD_ICD-11_ among refugees exists, but two systematic reviews have focused on cPTSD [13, 14]. Mellor et al. [13] included studies that assessed cPTSD as well as the precursor formulations EPCACE and disorder of extreme stress not otherwise specified (DESNOS) [15] from DSM-IV in refugees and other displaced persons. Nineteen studies were identified of which 10 used cPTSD criteria. The prevalence of cPTSD ranged from 3 to 51% and was associated with the number of PTE, trauma types including interpersonal violence, and postmigration living difficulties. Depression, anxiety, and somatization were the most frequent comorbidities. In an updated systematic review, da Silva et al. [14] again investigated cPTSD and the precursor formulations with a stronger focus on refugees and asylum seekers and validated or standardized diagnostic methods. Of the 15 included studies, 10 measured cPTSD and reported similar prevalence rates between 21.3 and 66.9% for treatment-seeking groups and between 3 and 50.9% for population-based groups.

The ICD-11 section of disorders specifically associated with stress also includes prolonged grief disorder (PGD_ICD-11_), a new diagnosis that is characterized by persistent longing for a deceased loved one or persistent preoccupation with the deceased [7]. These core symptoms are accompanied by intense emotional pain, and the grief reaction must persist for at least six months after the loss [7]. ICD-11 also includes a cultural caveat, stating that the grief reaction must exceed “expected social, cultural, or religious norms for the individual’s culture and context” [7]. PGD has also been included in the text revision of the DSM-5 (DSM-5-TR) [16], with slight differences in the diagnostic criteria. Four other criteria sets for prolonged grief exist as precursors to the current criteria. In 2009, consensus criteria were published by Prigerson et al. ([17]; PGD_Prigerson et al., 2009_). They required presence of a core symptom of separation distress, and five of nine additional cognitive, emotional, and behavioural symptoms with a time criterion of six months after the death. These criteria are a direct precursor to the ICD-11 criteria. The beta draft for ICD-11 [18] then defined persistent and pervasive yearning or longing, or persistent preoccupation with the deceased that persist for at least six months after the death as core symptoms for PGD and listed five additional symptoms that are also part of the current ICD-11 criteria. DSM-5 first proposed persistent complex bereavement disorder (PCBD) with a time criterion of 12 months [10]. Diagnosis required one of four symptoms of separation distress and six of 12 symptoms of reactive distress or social/identity disruption. An alternative criteria set was proposed by Shear and colleagues [19] called complicated grief (CG). Diagnosis of CG required one of four symptoms of separation distress, and two of eight accompanying symptoms with a time criterion of six months. All criteria sets share a definition of relatively similar core symptoms and differ in the number and content of accompanying symptoms and the time criterion. These differences have resulted in diverging prevalence rates [20, 21] but there is also a substantial overlap [22].

To date, one meta-analysis has investigated the prevalence and correlates of PGD in bereaved refugees, asylum seekers, and immigrants [23]. The analysis included 12 studies and yielded a pooled prevalence estimate of 33.2% (95% CI: 15.2–54.2%). There were moderate correlations between symptoms of PGD and PTSD and depression, respectively, as well as a concurrence of anxiety and somatic symptoms [23]. Predictors for PGD were having lost a first-degree relative, multiple and traumatic deaths, lack of rituals, female gender (for comorbid PGD and PTSD only), and older age. Higher education emerged as a protective factor.

Since the ICD-11 was released, the number of studies on the newly included and revised disorders has grown rapidly. The two most recent reviews that focus on refugees [14, 23] included studies published up to 2018 and 2020 respectively, and therefore an update is warranted. In addition, no review to date has focused jointly on PTSD, cPTSD, and PGD in refugees although the disorders appear in the same ICD-11 section and share the inclusion of an event criterion that can overlap in the case of traumatic deaths. The existing reviews [13, 23] indicate comorbidities among the disorders, as well as shared comorbidities with other mental health problems, and highlight the impact of PTE on cPTSD and PGD. The ecological model of refugee distress [24] proposes that refugee mental health is not only affected by prior exposure to PTE but also by ongoing postmigration living difficulties. These include all hardships related to migration that refugees face in their host country, including problems with the asylum procedure, housing difficulties, unemployment, separation from family, or discrimination. These stressors can have a substantial impact because they are daily stressors over which refugees have little or no control [24], and there is evidence that they contribute to mental health problems including stress-related disorders [13, 25, 26].

We therefore conducted a systematic review that investigated PTSD_ICD-11_, cPTSD, and PGD among refugees. The objective of this review was to examine (1) the prevalence of PGD, PTSD_ICD-11_, and cPTSD; (2) comorbidities; and (3) factors that predict or are associated with PGD, PTSD _ICD-11_, and cPTSD.

## Methods

### Inclusion criteria

This systematic review included quantitative studies published in peer-reviewed journals in English or German. Eligible studies had to study refugee and/or asylum seeker populations and report prevalence rates, predictors or associated factors, and/or comorbid mental disorders of at least one of the disorders PTSD_ICD-11_, cPTSD, or PGD. For PTSD and cPTSD, the studies had to adopt ICD-11 criteria because of the substantial differences in diagnostic criteria or the non-existence of the diagnosis in ICD-10 and DSM-5. For PGD, studies that followed ICD-11 criteria were eligible as well as studies that adopted the DSM-5-TR or earlier criteria sets. Given that PGD is a relatively new diagnosis, we wanted to include all potentially relevant studies, and recognise that there is substantial overlap between the criteria sets [22]. The review was not registered.

### Search strategy

The initial database search was conducted between June 2020 and April 2021 and the final search was run in November 2022. PubMed, Web of Science, and PsycArticles databases were systematically searched to identify potentially relevant articles (see Supplementary Table 1 for English search terms) .

### Study selection and data extraction

**Fig. 1:**
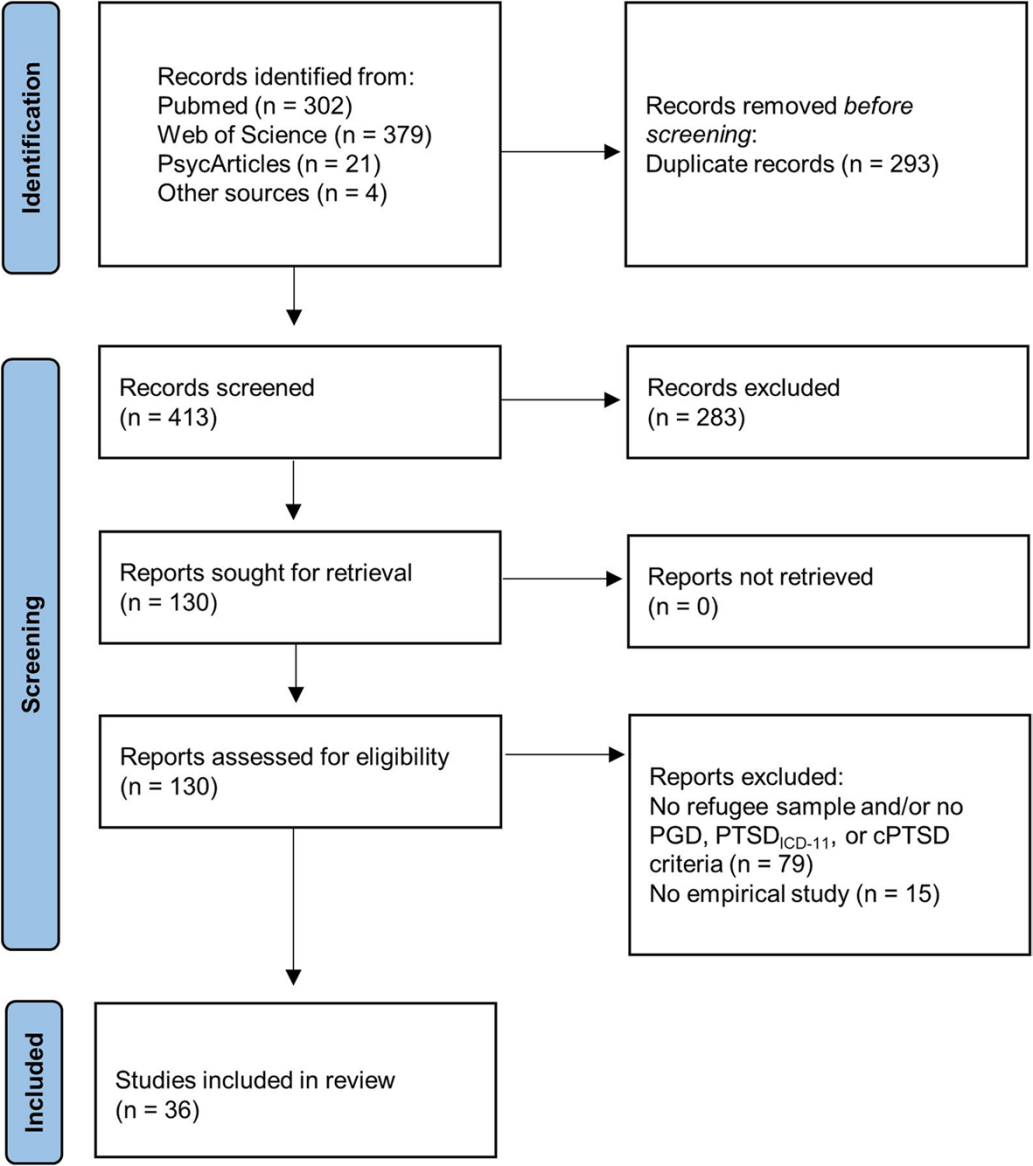
PRISMA flow diagram of study identification and selection

The study selection process followed the Preferred Reporting Items for Systematic Reviews and Meta-Analyses (PRISMA) guidelines ([27], Fig. 1). Identified articles were imported into the reference management program Zotero and were screened by title and abstract by one reviewer, with the removal of duplicates. Based on the full text, one reviewer assessed the study eligibility and when unsure, a second reviewer conducted a second screening. For all studies that met the inclusion criteria, the following data were then extracted into an Excel sheet: (1) study characteristics: names of the authors, year of publication, study design, outcome variables, and assessment instruments; (2) sample characteristics: country of origin, host country, sample size, gender, mean age; (3) prevalence rates, predictors and associated factors, and comorbid disorders; and (4) further results in line with the purpose of this review (e.g., frequency of PTE, number of lost loved ones).

### Study quality

Study quality was assessed independently by two reviewers. In case of disagreement, a third reviewer assessed the study and consensus was reached through discussion. We followed the approach used in a previous review and meta-analysis on PGD after violent loss [28]. For each study, 10 items (Supplementary Table 2) were rated as high risk of bias (score = 0), low risk of bias (score = 2), or unclear risk of bias (score = 1). Overall, scores ≥ 13 indicate high quality, scores between 10 and 12 indicate medium quality, and scores < 10 indicate low quality.

### Data synthesis

Data from the included studies were synthesized and a narrative summary was produced. We chose narrative synthesis over a more in-depth meta-analysis due to the small number of studies per variable of interest, heterogeneous samples, and heterogeneous outcomes, especially for PGD. For PTSD_ICD-11_ and cPTSD the pooled prevalence was calculated as a sample-size-weighted prevalence estimate. Due to different criteria sets for PGD and the small number of studies per criteria set, no pooled prevalence was calculated for PGD.

## Results

### Study characteristics

Thirty-six studies met the inclusion criteria. They were published between 2008 and 2022, with the majority (*n* = 30, 83.3%) published after 2015. All studies used cross-sectional methodologies and assessed refugees in 10 high-income (Austria, Australia, Belgium, Denmark, France, Germany, Italy, Switzerland, United States, and the United Kingdom) and four lower- and middle-income host countries (Jordan, Lebanon, Niger, and Papua New Guinea). Sixteen studies were conducted with individuals seeking or receiving treatment for mental health problems (i.e., treatment-seeking samples), nine studies were conducted with representative samples of refugees or refugee sub-samples from representative studies, and the remainder were conducted with convenience samples (i.e., population samples). All studies were published in English.

### Risk of bias

The results of the quality assessment are presented in Supplementary Table 2. Twenty-four studies (66.67%) were of high quality (score ≥ 13), nine studies (25%) were of medium quality (score = 10–12), and three studies (8.3%) were of low quality (score < 10). Overall, the studies included satisfactory information related to their objectives, study setting, eligibility criteria, and recruitment procedures, and provided sufficient descriptions of demographic and clinical characteristics. However, only three studies performed power calculations and described the proportions and handling of missing data.

### PGD

PGD was addressed in 18 studies that analysed 13 different samples (see Supplementary Table 3). Six studies investigated bereaved refugees, and across the other 12 studies between 28 and 92% of the participants reported the death of a loved one. The most frequently used instrument to assess symptoms of PGD (*n* = 5, see Supplementary Table 3) was the Inventory of Complicated Grief (ICG; [29]).

#### Prevalence

**Fig. 2:**
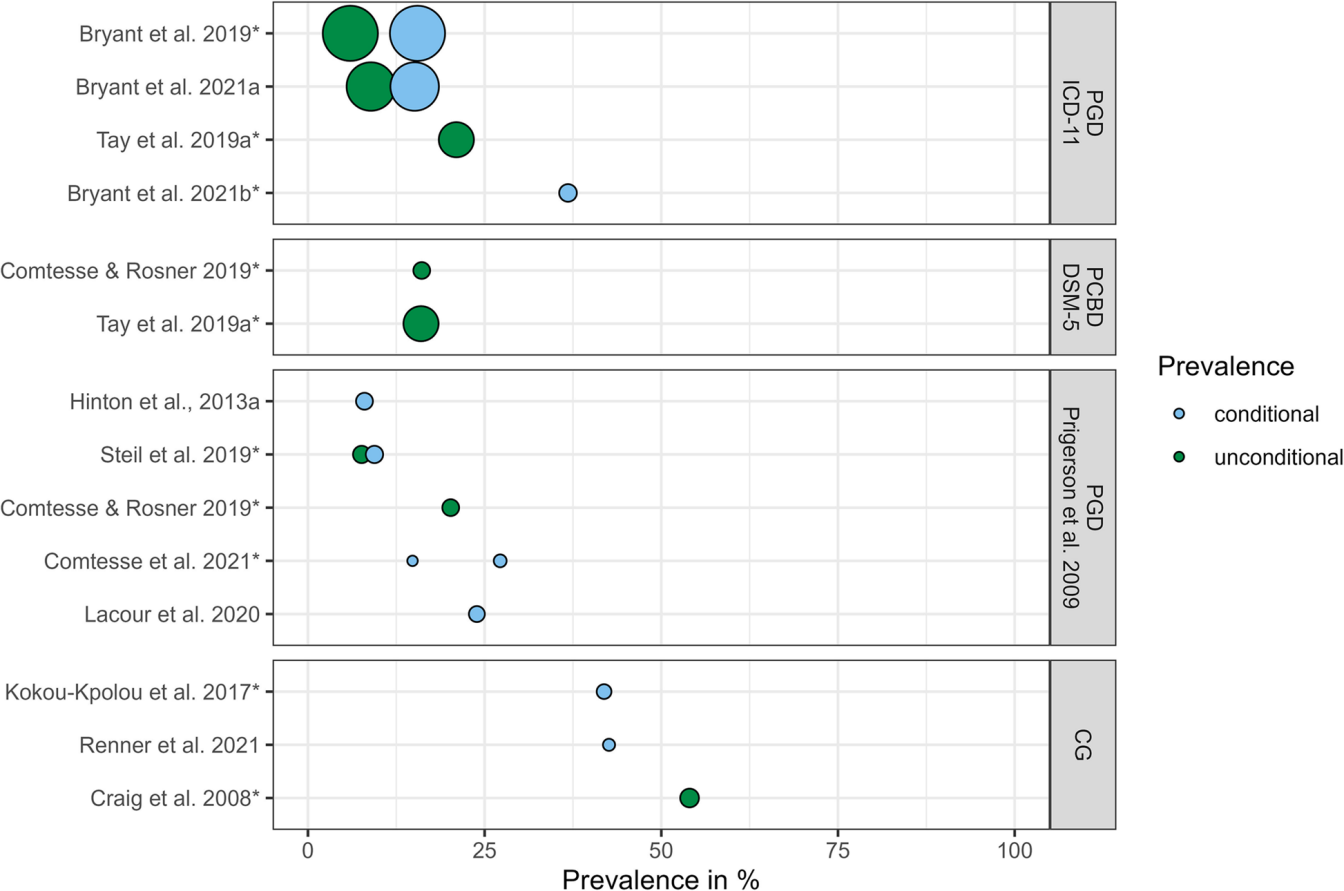
Prevalence rates for PGD per criteria set. Conditional prevalence = prevalence in bereaved (sub)samples, unconditional prevalence = prevalence in samples including bereaved and non-bereaved individuals. Larger circles represent larger sample sizes. Asterisks denote population samples

Twelve studies reported prevalence rates (Fig. 2). Four studies [30–33] used PGD_ICD-11_ criteria but the majority of studies (*n* = 5; [34–38]) used the precursor criteria PGD_Prigerson et al., 2009_. Three studies [39–41] referred to CG criteria, and *n* = 2 studies [33, 35] used PCBD criteria (see Supplementary Table 3). Overall, the prevalence of PGD ranged from 8 to 42.6% in bereaved refugees (i.e., conditional prevalence; [30, 32, 34–38, 40]) and from 6 to 54% in general samples including bereaved and non-bereaved individuals (i.e., unconditional prevalence; [30, 31, 33, 35, 38, 39]). Differences were observed depending on the applied criteria set. For PGD_ICD-11_ the unconditional prevalence ranged between 6 and 21% [30, 31, 33] and the conditional prevalence ranged between 15.1 and 36.8% [30–32]. For PGD_Prigerson et al., 2009_ the unconditional prevalence ranged between 7.6 and 20.2% [35, 38], and the conditional prevalence ranged between 8 and 23.9% [35–38]. The two studies that used PCBD criteria found unconditional prevalence rates of 16% [33] and 16.1% [35]. Notably, the group included in research conducted by Comtesse and Rosner [35] was composed almost entirely (92%) of bereaved refugees. The prevalence of CG was determined based on cut-off scores on the ICG. An unconditional prevalence of 54% with a score of 25 or greater [39] and a conditional prevalence rate of 41.9% with a score of 31 or greater [40] were reported. Two studies investigated prolonged grief after the disappearance of a loved one and found prevalence rates of 42.6% (scoring 25 or greater on the ICG; [41]) and 27.2% (according to PGD_Prigerson et al., 2009_; [34]).

#### Comorbid disorders and mental health correlates

Seven studies reported comorbid disorders or correlations between symptoms of PGD and other mental disorders. Comtesse and Rosner [35] found comorbid PTSD in 44% and comorbid depression in 11% of individuals; 38% fulfilled criteria for both PTSD and depression. Refugees with PGD in a population study in Australia [30] were also more likely to report probable PTSD and severe mental illness than those without PGD. Grief-related functional impairment was reported in 59% of participants in a study of Cambodian refugees living in the US [42].

PGD symptoms were highly correlated with PTSD symptoms according to DSM-IV, depressive symptoms, and anxiety in a sample of Bosnian refugees in the United States [39]. Well-being and general mental health were associated with fewer PGD symptoms in the same study [39]. Other studies found low [43] to moderate [44] correlations between symptoms of PGD and PTSD/cPTSD. A study with female refugees in Germany reported moderate correlations between PGD and depressive symptoms, anxiety, and somatization [38].

Two studies [33, 45] used latent class analysis (LCA) to elucidate symptom profiles and found a distinct PGD class (17%, 11%, of participants respectively) and a combined PTSD/PGD class (16%, 10%, of participants respectively). One of these studies, conducted with bereaved refugees in Australia [45], found that 75% of refugees in the PGD class and 97.4% of refugees in the PTSD/PGD class had comorbid depression.

#### Factors associated with PGD symptoms

Fourteen studies reported on factors associated with PGD symptoms, which were grouped into three broad categories: sociodemographic variables, trauma and loss characteristics, and postmigration living difficulties.

Six studies identified sociodemographic variables that were associated with PGD symptoms. Female gender was associated with higher PGD symptom severity [30, 31, 39], and emerged as a predictor for combined symptoms of PTSD and PGD [45]. No gender differences were identified in five studies [34, 35, 37, 40, 46, 47]. Older refugees tended to have higher PGD symptom severity in two studies [31, 39], and older age was a predictor for PGD class membership compared to resilient class membership in a representative sample of Mandaean refugees in Australia [45], but there was no association with age in six samples [34, 35, 37, 38, 40, 46, 47]. Differences in PGD symptom severity depending on religion and family status were found for female refugees [38]. For Togolese refugees, Kokou-Kpolou et al. [40] found that living in a couple was associated with higher symptom severity but religious affiliation was not. In the same study, refugees with a lower level of education also had higher PGD symptom severity [40]. Marital status did not play a role in four studies [30, 32, 37, 46] and there was no association with level of education in four studies [32, 35, 38, e who lost nuclear or close family members in three studi39].

Eleven studies reported associations with characteristics of loss and trauma. The relationship to the deceased influenced PGD symptoms in three studies: Higher PGD symptoms were observed in those who lost nuclear or close family members in three studies [35, 40, 41]. Relationship to the deceased did not play a role in a sample of Syrian refugees in a refugee camp in Jordan [32].

Higher exposure to PTE in general [38], and torture and combat exposure [30] as well as conflict-related PTE [47] specifically, predicted PGD symptoms. Experience of detention and abuse was a predictor of membership in both the PTSD/PGD and PGD class in one LCA [45]. There were no associations between the number of PTE and PGD symptomatology in three samples [34, 35, 37, 38].

Traumatic circumstances of the death was also a predictor of PGD symptoms [47] and combined PTSD and PGD symptoms [33, 45]. Bryant et al. [30] found that the murder and disappearance of family members was associated with PGD symptoms in a heterogenous sample of refugees in Australia, while there was no association with cause of death in a treatment-seeking sample of Syrian refugees in Jordan [32]. Unexpected death and no participation in bereavement rituals were associated with higher symptom severity in Togolese refugees [40]. Tay et al. [47] found that a sense of injustice was associated with PGD among West Papuan refugees.

Studies that investigated prolonged grief after the disappearance of a loved one found that ambiguous relative to confirmed loss led to higher symptom severity [34] and that a higher uncertainty about who belongs to the family system (i.e., boundary ambiguity) was associated with higher symptom severity [41]. Hinton et al. [36, 42] investigated specific grief-related predictors and found relationships with avoidance of reminders, rebirth concerns, and frequency of dreams about the deceased. Lacour et al. [37] found that lower perceived self-efficacy and difficulties in emotion regulation predicted PGD symptoms.

Six studies found evidence for an influence between postmigration living difficulties and PGD. The presence of postmigration living difficulties in general predicted membership in the PTSD/complicated bereavement class in West Papuan refugees [33] and adaptation difficulties were a predictor of membership in the PGD class in bereaved Mandaean refugees in Australia [45]. Discrimination also predicted PGD symptoms in a study among a heterogeneous sample of refugees in Australia [30].

Refugee status and length of stay over 10 years was associated with higher PGD severity in Togolese refugees living in Belgium and France [40]. Comtesse and Rosner [35] found that having a temporary residence permit (in comparison with waiting for asylum decisions and undergoing an appeal process) was associated with lower PGD symptoms in refugees in Germany. Steil et al. [38] found no association with application for asylum among a group of female refugees in Germany. Bryant et al. [30] found that refugees who were unemployed or received government benefits in Australia had higher PGD symptoms, whereas Tay et al. [46] found no association with employment in Papua New Guinea.

A lack of social support predicted PGD symptoms among a group of refugees in Germany [34]. Tay et al. [33] identified a relationship between PGD symptoms and disruptions of role and identity and the erosion of interpersonal bonds and networks. The loss of culture and support predicted combined PGD and PSTD symptoms in bereaved refugees in Australia [45]. Two other studies [37, 47] found no association with postmigration living difficulties.

### PTSD_ICD-11_ and cPTSD

PTSD_ICD-11_ and/or cPTSD were assessed in 19 studies that analysed 15 different samples (Supplementary Table 4). All studies, except for one [48], focused on both disorders. Most studies used the International Trauma Questionnaire (ITQ; [49]) to assess PTSD_ICD-11_ (*n* = 10) and cPTSD (*n* = 12). Other studies that used instruments that were not specifically developed to assess PTSD_ICD-11_ and cPTSD matched questionnaire items to the respective ICD-11 criteria (see Supplementary Table 4). The majority of studies studied traumatized groups of refugees, and exposure to PTE was high in other studies, with 56 [50] to 70.8% [51] of participating refugees reporting exposure to at least one PTE.

#### Prevalence

**Fig. 3:**
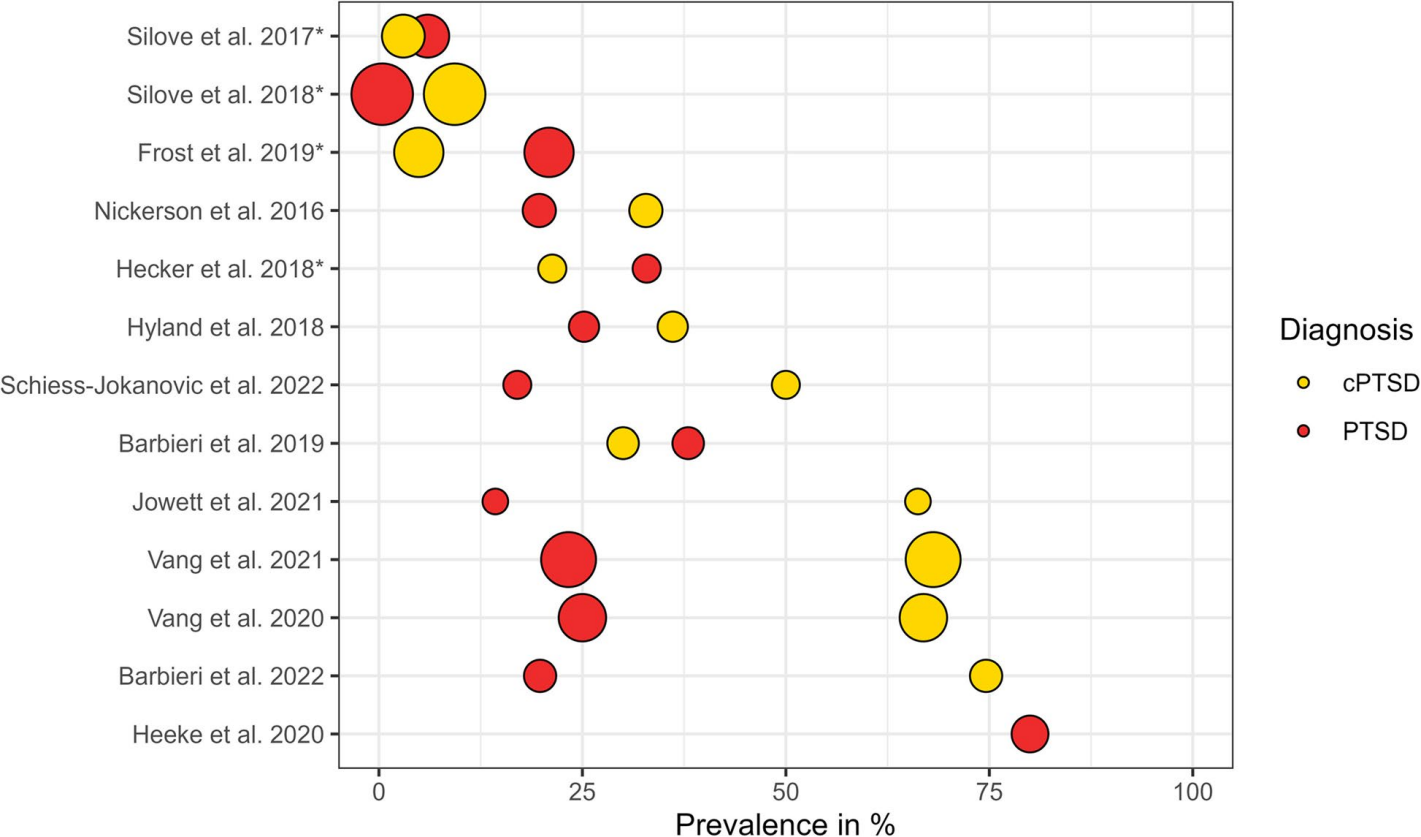
Prevalence rates of PTSD and cPTSD. Larger circles represent larger sample sizes. Asterisks denote population samples

Thirteen studies reported PTSD_ICD-11_ prevalence rates (Fig. 3), which ranged from 0.4 [52] to 80% [48]. The pooled prevalence of PTSD_ICD-11_ was 29.8% in treatment-seeking samples and 9.92% in population samples.

Twelve studies reported prevalence rates for cPTSD (Fig. 3) ranging from 3 [43] to 74.6% [53]. The pooled cPTSD prevalence was 57.4% in treatment-seeking samples and 7.8% in population samples.

#### Comorbid disorders and mental health correlates

Comorbidities and mental health correlates of PTSD_ICD-11_ and cPTSD symptoms were reported in nine studies. Silove et al. [52] found two or more common mental disorders in 97.8% of West Papuan refugees. In a group in Switzerland [54], the risk of developing depressive episodes was higher in 26 and 80% of refugees with PTSD_ICD-11_ and cPTSD, respectively. Heeke et al. [48] reported comorbid depression in almost the entire group of refugees in a specialized treatment center (99%) and also found a high comorbidity with anxiety (97%). Vang et al. [55] reported low associations between PTSD_ICD-11_ and depression and high associations between DSO and PTSD_ICD-11_, depression, and anxiety in Syrian refugees in Denmark. Silove et al. [43] found moderate correlations between symptoms of depression and PTSD_ICD-11_ and cPTSD for West Papua refugees in Papa New Guinea.

The levels of functional impairment regarding social life, work, or other important areas of life were high in refuges with PTSD_ICD-11_ [56] and cPTSD [53, 56]. West Papuan refugees with cPTSD had greater impairment in six domains (i.e., understanding and communicating, mobility, self-care, getting along, life activities, and participation in society) than refugees with another common mental disorder or without a disorder [52, 57]. In a sample of treatment-seeking refugees in the UK [58], those with PTSD_ICD-11_ and cPTSD had higher healthcare needs than those without these diagnoses, and those with cPTSD had greater levels of distress and higher relationship-related needs.

LCA and cluster analysis have been used in eight studies to investigate if homogeneous subgroups with PTSD_ICD-11_ and cPTSD symptoms exist [51, 53, 56, 59–63]. Studies found distinct PTSD_ICD-11_ (13% [59] to 44.44% [63] of participants) and cPTSD classes (13.3% [51] to 87% [59]). Liddell et al. [62] also found an affective dysregulation class (31.9%) and Frost et al. [51] found a PTSD_ICD-11_ low mood class (3.6%).

#### Factors associated with PTSD and cPTSD

Eleven studies reported factors that were associated with PTSD_ICD-11_ and cPTSD symptoms, which we grouped into sociodemographic variables, trauma characteristics, and postmigration living difficulties.

Five studies identified sociodemographic variables associated with PTSD_ICD-11_ and cPTSD. Female gender was a predictor of PTSD_ICD-11_ [55, 62] and cPTSD [51, 62]. Eight studies did not establish gender differences [53–56, 58–60, 64].

Vang et al. [55] found that increasing age predicted PTSD_ICD-11_ symptoms but not DSO symptoms, and older homeland born West Papuan refugees also had higher PTSD_ICD-11_ symptoms [50]. There was no association with age in five other studies [53, 56, 59, 60, 64]. More months spent in the host country Italy predicted the severity of PTSD_ICD-11_ but not class membership [60]. More months spent in the host country Lebanon did also not predict class membership among treatment-seeking Syrian refugees [56].

Several studies found evidence for an association with trauma load and type. A higher number of PTE predicted PTSD_ICD-11_ class membership [62] and PTSD_ICD-11_ symptom severity [54]. More specifically, Frost [51] reported that the experience of being an unarmed civilian during war, revolution, or a military coup, and experiencing a serious accident were predictors of PTSD_ICD-11_ class membership. Accordingly, cPTSD was also associated with a higher number of PTE [51, 52, 57, 62] and there was an association with childhood trauma in refugees living in Niger [53] and Afghan refugees [63]. More childhood adversities were found for refugees with cPTSD than for those with PTSD_ICD-11_ in West Papuan refugees [52]. Frost et al. [51] found that physical assault, neglect, sexual assault, and serious accidents were predictors of cPTSD class membership. Three studies [53, 55, 60] found no association with the number of experienced PTE, and seven studies [56, 58, 59, 61–63, 65] found no associations with trauma types.

Associations with postmigration living difficulties were reported in six studies almost exclusively for DSO and cPTSD. A lack of social support and postmigration living difficulties in general predicted DSO but not PTSD_ICD-11_ symptoms in refugees living in Switzerland [54]. In a network analysis [66], discrimination and socio-economical living conditions, language acquisition and barriers, and residence insecurity were connected to cPTSD symptom clusters. Silove et al. [52] and Tay et al. [57] found more postmigration living difficulties in West Papuan refugees with cPTSD than in those with a common mental disorder and without a disorder. In a sample of refugees living in Niger, those with cPTSD were more likely to live in a large reception facility in the desert than in small urban facilities [53]. Insecure visa status predicted PTSD_ICD-11_ and cPTSD class membership in refugees in Australia [62]. In Afghan refugees in Austria, the cPTSD cluster was associated with more problems with language acquisition and barriers than the PTSD_ICD-11_ cluster while the extent of other postmigration living difficulties did not differ between the clusters [63]. There were no associations between PTSD_ICD-11_ or cPTSD and employment [53, 56, 59, 60], family concerns [66], legal status [60], and being with family [53].

## Discussion

This systematic review investigated the prevalence of PGD, PTSD_ICD-11_ and cPTSD, their comorbidities, and factors that predict or are associated with these disorders in adult refugees in countries with different income levels. Prevalence rates ranged from 6 to 54% for prolonged grief, 0.4 to 80% for PTSD_ICD-11_, and 3 to 74.6% for cPTSD. The pooled prevalence of PTSD_ICD-11_ and cPTSD was 29.8 and 57.4% in treatment seeking samples and 9.92 and 7.8% in population samples. Depressive symptoms were the most common comorbidity across disorders, and PTSD often co-occurred with PGD. Several sociodemographic variables, trauma and loss characteristics, and postmigration living difficulties were associated with symptom severity.

Most of the 36 included studies were of high quality, and only 8.3% were of low quality. The most common shortcomings were missing information regarding power calculations and missing data, and not using probability sampling.

### Prevalence

The range of reported prevalence rates for PGD was extremely wide (i.e., 6–54%). Previous meta-analyses have yielded a PGD prevalence of 9.8% in bereaved non-refugee samples after mainly non-violent losses [67] and a much higher pooled prevalence of 49% in non-refugee and conflict-affected groups after unnatural deaths [68]. The meta-analysis by Kokou-Kpolou et al. [23] found a pooled prevalence of 33.2% among refugees that falls into the range found in the present review. Of the four studies included in that meta-analysis, three [23, 36, 39] were also included in the present review (the fourth was excluded because it did not study refugees but conflict survivors).

The included studies used four different criteria sets for PGD to determine prevalence rates, which partly explains the wide range. This heterogeneity reflects the development of diagnostic criteria for PGD but makes it difficult to compare results. Only four of the 12 studies that reported PGD prevalence rates applied ICD-11 criteria. A recent population-based study in Germany found a conditional prevalence of 4.2% and an unconditional prevalence of 1.5% for PGD_ICD-11_ [69]. In the present review, the unconditional prevalence for PGD_ICD-11_ ranged between 6 and 21%, and the conditional prevalence ranged between 15.1 and 36.8%, which corroborates a much higher prevalence among refugees [23]. The highest prevalence rates were reported in studies that used CG criteria. This pattern is comparable to the differences between criteria sets that have been reported previously (e.g. [22, 70]). No study used DSM-5-TR criteria, which were officially released only seven months before the last literature search.

There is also a growing body of literature that has investigated the prevalence of PTSD_ICD-11_ and cPTSD in various groups [71]. Prevalence rates ranged from 0.6 to 1% for cPTSD and from 2.3 to 3% for PTSD_ICD-11_ in community and representative groups [72, 73], and from 42.8 to 53.1% for cPTSD and 7.8–37% for PTSD_ICD-11_ in treatment-seeking groups [73, 74]. The prevalence rates in the studies included in the present review were much higher. We also found higher prevalence rates for cPTSD than for PTSD_ICD-11_ in treatment-seeking samples, which corresponds with the higher functional impairment that we found associated with cPTSD. The prevalence rates we identified for cPTSD were also higher than those reported in previous reviews of refugees and displaced persons (3–51%; [13], 3–66.9%; [14]). Our review included six [50–52, 54, 56, 65] and nine studies [50–52, 54, 56, 59, 60, 62, 65] that were also included in the reviews by Mellor et al. [13] and de Silva et al. [14], respectively. Recent studies included in the present review reported some of the highest prevalence rates in mostly treatment-seeking groups [48, 53, 55, 58, 66].

The highest PTSD_ICD-11_ prevalence was found in a study with a group seeking treatment from a specialized treatment center for refugees in Germany [48]. It is possible that this specialization attracted more severe cases. It is also the only study that investigated PTSD_ICD-11_ but not cPTSD. It is very likely that some patients met criteria for cPTSD and the prevalence of PTSD_ICD-11_ would have been lower if cPTSD had also been diagnosed (i.e., because patients who meet diagnosis of cPTSD do not receive an additional diagnosis of PTSD_ICD-11_). The lowest PTSD_ICD-11_ prevalence rates were reported in two representative samples of West Papuan refugees in Papua New Guinea [43, 52]. Almost all participants in the study by Silove et al. [52] who met the PTSD_ICD-11_ criteria also met the DSO criteria and therefore received a diagnosis of cPTSD (0.4% PTSD_ICD-11_, 9.3% cPTSD). In the second study [43], the prevalence of cPTSD was lower than the prevalence of PTSD (6% PTSD_ICD-11_, 3% cPTSD), and also the lowest of all the included studies. This may be explained by a shorter duration of flight for this group or the general finding that PTSD prevalence tends to be lower in more vulnerable countries according to social and economic features [75]. However, prevalence rates did not differ between treatment-seeking groups in high- income and low- and middle-income countries; the highest prevalence of cPTSD (74.6%) was reported in a study of refugees in a humanitarian setting in Niger [53]. All study participants were treatment-seeking and experienced uncertainty regarding their visa status, which repeatedly emerged as an important influence on symptom severity.

### Comorbidities

The most frequent comorbidities of PGD were PTSD and depression. This is in line with consistent findings that some bereaved persons develop symptoms of both PGD and PTSD when the loss occurs under traumatic circumstances [28, 76, 77]. The co-occurrence of PGD and PTSD symptoms may be even more pronounced in refugees who often experience traumatic losses. Depressive symptoms have also been shown to co-occur with PGD symptoms in a meta-analysis of PGD after traumatic loss [28], and in a LCA of people bereaved by unnatural losses 35.5% had combined PGD and depressive symptoms [78]. The studies in the present review also reported correlations with somatization and anxiety. Somatic symptoms appear to be important in culturally diverse groups as an expression of emotional distress [79].

Depression was the most common comorbid disorder for PTSD_ICD-11_ and cPTSD, followed by anxiety. CPTSD symptoms were also associated with more functional impairment in various domains of life than PTSD_ICD-11_ symptoms. A representative study from the United Kingdom [80] also found high levels of comorbidity with depression, and participants with cPTSD were more likely to have depression and generalized anxiety disorder compared to participants with PTSD_ICD-11_. Higher functional impairment in cPTSD than in PTSD_ICD-11_ has been consistently reported (e.g., [71, 81, 82]). It is not surprising that the additional DSO symptoms such as difficulties in sustaining and building relationships or regulating affective states lead to more serious problems in professional and private contexts.

PTSD is often the focus of research and treatment of refugee mental health problems. The high prevalence rates of PTSD_ICD-11_ support this approach, but the focus needs to be extended to cPTSD. There is evidence for effective intervention programs from non-refugee studies [83]. Studies with refugees that focused on interventions for cPTSD or used culturally sensitive interventions also show promising results [84, 85]. The present results also highlight that bereaved refugees often suffer from PGD, alone or in combination with PTSD_ICD-11_ symptoms. The high prevalence rates underline the necessity of extending mental health screenings of refugees to include PGD and to provide subsequent specialized interventions. This is also important because there is evidence that PGD symptoms in the first year after bereavement may contribute to the exacerbation of PTSD symptoms [86]. To date, only a few studies have investigated PGD treatment for refugees [87] and future studies need to examine with controlled designs how far the positive findings for PGD treatment in non-refugee populations [88] are generalizable to refugee groups.

### Associated factors

Our results regarding associations of PGD, PTSD_ICD-11_, and cPTSD with sociodemographic variables and characteristics of trauma and loss are generally in line with findings from non-refugee groups. As in previous studies, we found some evidence that females are more at risk for PGD [23, 89] and PTSD [90], but the results remain inconsistent. Older age was identified as a moderator of PGD symptoms in a meta-analysis of prolonged grief after mainly non-violent loss [67], but there was more evidence against a relationship between PGD symptoms and age in our review. However, the age range was also narrower in studies included in our review.

For PGD, results are in line with the consistent finding that a closer relationship with the deceased puts an individual at greater risk [28, 89]. The role of traumatic circumstances of a death in the development of PGD has also been previously identified for refugee [23] and non-refugee groups [68]. The importance of ambiguous loss after the disappearance of a closed loved one and uncertainty regarding their fate also emerged repeatedly. Although a diagnosis of PGD cannot be made after a disappearance, the findings on ambiguous loss have implications for mental health care for refugees. In Syria alone, an estimated 102,000 people have forcibly disappeared since 2011 [91]. Clinicians therefore need to be aware of possible PGD symptoms resulting from the disappearance of loved ones, screen for it with specialized measures [92], and address it with interventions focused on dealing with the persistent uncertainty regarding the whereabouts of the disappeared person [93].

Exposure to PTE other than traumatic circumstances of a death also emerged as a consistent risk factor. This is in line with findings that PTE are associated with worse mental health beyond PTSD in different groups including refugees [94]. Given that refugees are confronted with numerous PTE [2], a (traumatic) death may often happen in the context of other PTE and the resulting stress may increase vulnerability for the development of PGD. Screening for losses and their circumstances in combination with trauma history is therefore necessary. Factors associated with PTSD and cPTSD are also in line with previous research regarding the influence of trauma load [90] and the role of childhood trauma especially in cPTSD [71, 80].

The third category of associated factors, postmigration living difficulties, is unique to refugees and lends further evidence to the ecological model of refugee distress [24]. It is likely that insecure visa status and uncertainty impact feelings of safety, and language problems hinder social contacts, work opportunities, and access to mental health services which then influences mental health outcomes.

Although living circumstances and asylum policies in the host countries represented in the review differ, postmigration living difficulties were correlated with PGD symptoms across high- and low- and middle-income countries. Social support has been identified as one of the most important risk factors for PTSD in non-refugee traumatized groups [90], but more studies in the present review reported its correlation with PGD. In individualistic host societies, refugees often experience loneliness, which has negative effects on health [95, 96]. When the deceased was the major source of social support, building new relationships of trust and support may be especially difficult in a post-migration setting. Depending on the country of origin, bereavement rituals involving the larger community are also an important part of bereavement and a lack of shared mourning can hinder adaptation to the loss [97].

More evidence for associations with postmigration living difficulties were found for cPTSD than for PTSD_ICD-11_. It seems plausible that these ongoing stressors have a particularly detrimental effect on self-concept, functioning in relationships, and emotion regulation. However, our results do not allow us to infer causal relationships and it is also likely that DSO symptoms complicate adaptation to the host country, for example due to reduced capacities to learn a new language or succeed at a new workplace. Relationships like these may potentially create a vicious circle that needs to be broken by specific interventions and policy changes.

As postmigration living difficulties can hinder the delivery of trauma- and grief-focused interventions [98] and even limit their effects [87], the findings of our review call for multi-discipline interventions that provide support to refugees to deal with these stressors. Postmigration living difficulties can also be addressed in interventions by incorporating problem solving interventions that can help to manage stressors [99] or by providing learning opportunities and access to resources [100]. On a policy level, simpler and faster asylum procedures are needed [24].

### Limitations and future directions

The review is limited by drawing only from English-language studies. Due to the heterogeneity in the groups, contexts of assessment, and outcome measurement (for PGD), no meta-analysis was conducted. Further limitations stem from methodological shortcomings in the included studies.

The heterogeneity of criteria sets used to determine the prevalence of PGD made it difficult to synthesize results. The instrument used most often to assess PGD symptoms was the ICG, which does not follow current diagnostic criteria. While we expect that future studies will yield prevalence estimates derived from current conceptualizations of the disorder, it is also important that future studies assess potential risk factors that are specific to PGD. For example, only a few studies in the present review assessed the importance of being able to complete mourning rituals or the influence of avoidance behaviour that has been described as an important factor in the development and maintenance of PGD [101, 102].

ICD-11 was developed with a focus on international applicability. Against this background, it was reassuring to see that about half of the studies on PTSD_ICD-11_ and cPTSD were conducted with the ITQ that was developed consistent with ICD-11 principles. For PGD as well as PTSD_ICD-11_ and cPTSD, some studies also used instruments that were culturally sensitive [42] or adapted for specific refugee groups [33, 43, 46, 47, 50, 52]. Other studies are limited by their use of instruments that were developed with Western samples without including additional emic perspectives or performing cultural adaptations. Culturally-sensitive assessment is important because studies found cross-cultural variation in grief symptoms [79] and different PGD prevalence rates between cultures [103, 104]. This raises the question of comparability of the included studies both regarding intensity of the symptoms and types of symptoms reported. Future studies need to disentangle the effects of measurement and culture by focusing on measurement invariance of assessment instruments across cultures and then compare core symptomatology and possible additional culture-specific symptoms.

Although 83% of refugees are hosted in low- and middle-income countries [1], most of the included studies were conducted in high-income countries. More studies with refugees settled in low- and middle-income countries are needed, particularly in order to better understand and compare the influence of the full range of postmigration living difficulties. In addition, most studies also used treatment-seeking samples or convenience samples and are prone to sampling bias. It can also not be ruled out that some participants were motivated to participate because they expected positive consequences for asylum procedures or other postmigration living difficulties, which may have led to an overrepresentation of those refugees with more severe difficulties. Many studies were heterogeneous in terms of the studied refugee population, including differences regarding the country of origin, available health care in the host country, and policies that influence postmigration living difficulties. This heterogeneity hindered comparisons and precludes generalization of the results. There is also a need for longitudinal studies to unveil causal relationships between symptoms and the more variable factors such as social support and postmigration living difficulties. Given the high and increasing number of refugees these insights can provide valuable foundations for interventions and carry implications for policy changes.

## Conclusions

Given the heterogeneity of the included studies, ranges for prevalence rates were wide but a general pattern of high prevalence rates and comorbidities for PTSD_ICD-11,_ cPTSD, and PGD emerged. Evidence regarding factors associated with symptom severity were in line with previous research with non-refugee groups and also corroborate the detrimental relationship between postmigration living difficulties and psychopathology. The findings of this review highlight the importance of offering trauma- and grief-focused interventions to refugees. They also have implications for the design of interventions that take into account comorbidities especially after traumatic loss and the influence of postmigration living difficulties. The latter also calls for changes on a policy level.

### Supplementary Information

**Supplementary Material 1.**

**Supplementary Material 2.**

**Supplementary Material 3.**

**Supplementary Material 4.**

## Data Availability

The datasets used and/or analysed during the current study are available under https://osf.io/auz7q/(doi: 10.17605/OSF.IO/AUZ7Q).

## Abbreviations

AUDADIS-IV: Alcohol Use Disorder and Associated Disabilities Face-to-face-interview Schedule-DSM-IV Version
CG: Complicated grief
cPTSD: Complex posttraumatic stress disorder
DERS: Difficulties in emotion regulation scale
DESNOS: Disorder of extreme stress not otherwise specified
DSO: Disturbances in self-organization
DSM-5: Diagnostic and statistical manual of mental disorders, fifth edition
DSM-IV: Diagnostic and statistical manual of mental disorders, fourth edition
DSM-5-TR: Diagnostic and statistical manual of mental disorders, fifth edition, text revision
ECR: Experiences in close relationships scale
EPCAGE: Enduring personality change after catastrophic experience
HSCL: Hopkins symptom checklist
ICD-11: International statistical classification of diseases and related health problems, 11th revision
ICG: Inventory of complicated grief
ITQ: International trauma questionnaire
LCA: Latent class analysis
PCBD: Persistent complex bereavement disorder
PCL-5: PTSD checklist for DSM-5
PGD: Prolonged grief disorder
PG-13: Prolonged Grief-13
PDS: Posttraumatic diagnostic scale
PRISMA: Reporting items for systematic reviews and meta-analyses
PSS-I: PTSD symptom scale-face-to-face-interview version
PTSD: Posttraumatic stress disorder
R-MHAP: Refugee-mental health assessment package
SIDES-SR: Structured interview of disorders of extreme stress-self report
TGI-SR: Traumatic Grief Inventory
