# Supplementary material for: Prevalence, comorbidities, and factors associated with prolonged grief disorder, posttraumatic stress disorder and complex posttraumatic stress disorder in refugees: a systematic review
Source: Confl Health. 2024 Apr 16;18:32. doi: 10.1186/s13031-024-00586-5 (PMC11020800; doi:10.1186/s13031-024-00586-5)
Supplement: Supplementary file 1 — Supplementary Material 1. [file 13031_2024_586_MOESM1_ESM.docx]

Supplementary Table 1

*English search terms per database*

|  | PubMed | Web of Science | PsycArticles |
| --- | --- | --- | --- |
| Posttraumatic Stress Disorder | „(((Posttraumatic Stress Disorder) OR (PTSD) OR (Post Traumatic Stress Disorder)) AND ((Refugee*) AND ((ICD 11) OR (International Statistical Classification of Diseases 11)))” | „ALL = (((Posttraumatic Stress Disorder) OR (PTSD) OR (Post Traumatic Stress Disorder)) AND (Refugee*) AND ((ICD 11) OR (International Statistical Classification of Diseases 11)))” | „(((Any Field: Posttraumatic Stress Disorder) OR (Any Field: PTSD) OR (Any Field: Post Traumatic Stress Disorder)) AND (Any Field: Refugee*) AND ((Any Field: ICD 11) OR (Any Field: International Statistical Classification of Diseases 11)))” |
| Complex Posttraumatic Stress Disorder | „(((Complex Posttraumatic Stress Disorder) OR (Complex PTSD) OR (CPTSD) OR (Complex Post Traumatic Stress Disorder)) AND (Refugee*))” | „ALL = (((Complex Posttraumatic Stress Disorder) OR (Complex PTSD) OR (CPTSD) OR (Complex Post Traumatic Stress Disorder)) AND (Refugee*))” | „(((Any Field: Complex Posttraumatic Stress Disorder) OR (Any Field: Complex PTSD) OR (Any Field: CPTSD) OR (Any Field: Complex Post Traumatic Stress Disorder)) AND (Any Field: Refugee*))” |
| Prolonged Grief Disorder | „(((Prolonged Grief Disorder) OR (Prolonged Grief) OR (Complicated Grief) OR (Persistent Complex Bereavement Disorder) OR (PGD) OR (CG) OR (PCBD)) AND (Refugee*)” | „ALL = (((Prolonged Grief Disorder) OR (Prolonged Grief) OR (Complicated Grief) OR (Persistent Complex Bereavement Disorder) OR (PGD) OR (CG) OR (PCBD)) AND (Refugee*)))” | „(((Any Field: Prolonged Grief Disorder) OR (Any Field: Prolonged Grief) OR (Any Field: Complicated Grief) OR (Any field: Persistent Complex Bereavement Disorder) OR (Any Field: PGD) OR (Any field: CG) OR (Any field: PCBD)) AND ((Any Field: Refugee*)))” |
