# Supplementary material for: Prevalence, comorbidities, and factors associated with prolonged grief disorder, posttraumatic stress disorder and complex posttraumatic stress disorder in refugees: a systematic review
Source: Confl Health. 2024 Apr 16;18:32. doi: 10.1186/s13031-024-00586-5 (PMC11020800; doi:10.1186/s13031-024-00586-5)
Supplement: Supplementary file 2 — Supplementary Material 2. [file 13031_2024_586_MOESM2_ESM.docx]

Supplementary Table 2.

*Individual item-based rating of the quality of included studies.*

| Study | Study objectives | Eligibility criteria | Recruitment | Setting | Representative sample | Missing data | Power calculation | Demographic and clinical characteristics | Study limitations | Quality (sum) | Quality rating |
| --- | --- | --- | --- | --- | --- | --- | --- | --- | --- | --- | --- |
| Barbieri et al. (2019) | 2 | 2 | 2 | 2 | 1 | 1 | 0 | 2 | 2 | 14 | High |
| Barbieri et al. (2022) | 2 | 2 | 2 | 2 | 2 | 0 | 0 | 2 | 2 | 14 | High |
| Bryant et al. (2019) | 2 | 2 | 2 | 2 | 2 | 1 | 0 | 2 | 2 | 15 | High |
| Bryant et al. (2020) | 2 | 2 | 2 | 2 | 2 | 2 | 0 | 2 | 2 | 16 | High |
| Bryant et al. (2021) | 2 | 2 | 2 | 2 | 0 | 0 | 0 | 1 | 0 | 9 | Low |
| Comtesse & Rosner (2019) | 2 | 2 | 2 | 2 | 0 | 2 | 0 | 2 | 2 | 14 | High |
| Comtesse et al. (2021) | 2 | 2 | 2 | 2 | 0 | 1 | 0 | 2 | 2 | 13 | High |
| Craig et al. (2008) | 2 | 1 | 1 | 2 | 2 | 2 | 2 | 2 | 2 | 16 | High |
| Frost et al. (2019) | 2 | 2 | 2 | 2 | 2 | 2 | 0 | 2 | 1 | 15 | High |
| Hecker et al. (2018) | 2 | 2 | 2 | 2 | 2 | 0 | 2 | 2 | 2 | 16 | High |
| Heeke et al. (2020) | 2 | 1 | 2 | 2 | 0 | 2 | 0 | 2 | 2 | 13 | High |
| Hinton et al. (2013a) | 1 | 1 | 2 | 1 | 0 | 0 | 0 | 1 | 1 | 7 | Low |
| Hinton et al. (2013b) | 2 | 2 | 1 | 1 | 0 | 0 | 0 | 1 | 2 | 9 | Low |
| Hyland et al. (2018) | 2 | 2 | 2 | 1 | 0 | 0 | 0 | 2 | 2 | 11 | Medium |
| Jowett et al. (2021) | 2 | 2 | 2 | 2 | 0 | 2 | 0 | 1 | 2 | 13 | High |
| Kokou-Kpolou et al. (2017) | 2 | 2 | 2 | 2 | 0 | 2 | 0 | 2 | 2 | 14 | High |
| Lacour et al. (2020) | 2 | 2 | 2 | 1 | 1 | 0 | 0 | 2 | 2 | 12 | Medium |
| Liddell et al. (2019) | 2 | 2 | 2 | 2 | 2 | 2 | 0 | 2 | 2 | 16 | High |
| Nickerson et al. (2011) | 2 | 2 | 2 | 2 | 0 | 0 | 1 | 2 | 2 | 13 | High |
| Nickerson et al. (2014) | 2 | 2 | 2 | 2 | 2 | 2 | 0 | 2 | 2 | 16 | High |
| Nickerson et al. (2016) | 2 | 2 | 2 | 2 | 0 | 2 | 0 | 2 | 2 | 14 | High |
| Palic et al. (2016) | 2 | 1 | 1 | 1 | 2 | 0 | 0 | 2 | 2 | 11 | Medium |
| Renner et al. (2021) | 2 | 2 | 1 | 2 | 0 | 0 | 0 | 2 | 2 | 11 | Medium |
| Schiess‑Jokanovic et al. (2021) | 2 | 2 | 1 | 1 | 0 | 2 | 1 | 0 | 2 | 11 | Medium |
| Schiess‑Jokanovic et al. (2022) | 2 | 2 | 0 | 1 | 0 | 2 | 0 | 2 | 2 | 11 | Medium |
| Silove et al. (2017) | 2 | 1 | 2 | 2 | 0 | 0 | 0 | 2 | 2 | 11 | Medium |
| Silove et al. (2018) | 2 | 2 | 2 | 2 | 2 | 2 | 0 | 2 | 2 | 16 | High |
| Steil et al. (2019) | 2 | 2 | 2 | 2 | 2 | 2 | 0 | 2 | 2 | 16 | High |
| Tay et al. (2015) | 2 | 1 | 2 | 2 | 0 | 0 | 0 | 2 | 2 | 11 | Medium |
| Tay et al. (2016) | 2 | 1 | 2 | 2 | 2 | 0 | 2 | 2 | 2 | 15 | High |
| Tay et al. (2018a) | 2 | 2 | 2 | 2 | 0 | 0 | 0 | 2 | 2 | 12 | Medium |
| Tay et al. (2018b) | 2 | 2 | 2 | 2 | 0 | 2 | 0 | 2 | 2 | 14 | High |
| Tay et al. (2019) | 2 | 2 | 2 | 2 | 0 | 2 | 0 | 2 | 2 | 14 | High |
| Vallières et al. (2018) | 2 | 2 | 2 | 2 | 0 | 2 | 0 | 2 | 2 | 14 | High |
| Vang et al. (2019) | 2 | 2 | 2 | 2 | 0 | 2 | 0 | 2 | 2 | 14 | High |
| Vang et al. (2021) | 2 | 2 | 1 | 2 | 0 | 1 | 0 | 1 | 2 | 11 | High |

Note. 0 = not reported/no, 1 = unclear, 2 = reported. Low quality = total score < 10; medium quality = total score 10-12; high quality = total score ≥ 13.
