# Supplementary material for: Prevalence, comorbidities, and factors associated with prolonged grief disorder, posttraumatic stress disorder and complex posttraumatic stress disorder in refugees: a systematic review
Source: Confl Health. 2024 Apr 16;18:32. doi: 10.1186/s13031-024-00586-5 (PMC11020800; doi:10.1186/s13031-024-00586-5)
Supplement: Supplementary file 3 — Supplementary Material 3. [file 13031_2024_586_MOESM3_ESM.docx]

Table 1.

*Study Characteristics and Main Findings for PGD*

| Study | Study design & data collection | Sample size & population type | Country of origin | Host country | Gender | Age | Outcome variables (instrument) | PGD criteria | Summary of main findings |
| --- | --- | --- | --- | --- | --- | --- | --- | --- | --- |
| Bryant et al. (2019) | Cross-sectional (wave 3 of a longitudinal population-based cohort study); Face-to-face-interview | *N* = 1783  (Adults: *n* = 1.245; Children: *n* = 538)  Nationally representative sample | Iraq/Afghanis-tan: 54.9%; Bhutan/Myan-mar: 17.1%; Iran: 8.1%; Libya/Syria/  Egypt: 6.9%; Pakistan: 6.1%;  Sub-Sahara Africa: 4.2%; Sri Lanka/India: 2.8% | Australia | Adults:  61.7% male;  Children: 53.4% male | Adults with PGD:  *M* = 45.9 (*SE* = 1.6)  Adults without PGD:  *M* = 38.8 (*SE* = 0.4)  Children: *M* = 11.6 (*SD* = 3.9) | PGD (4 items; Shear et al., 2011);  PTSD (PTSD-8); Trauma exposure (HTQ); Post-migration stressors (self-developed); Severe mental illness (K6); Children’s psychological problems (SDQ) | ICD-11 | - 39.4% reported bereavement - Prevalence PGD (ICD-11): 6.0%, (conditional prevalence 15.5%) - Comorbidities: PTSD, severe mental illness, more psychological difficulties in children of refugees with PGD than without PGD - Group differences: female gender; unemployment; received psychological help in Australia; murder/disappearance of family; torture; combat exposure; trauma exposure; discrimination; receiving government benefits - No association with years since arrival |
| Bryant et al. (2021b) | Longitudinal population-based cohort study; Face-to-face-interview | *N* = 288  (Adults: *n* = 110; Children: *n* = 178)  Bereaved sample | Iraq/Afghanistan: 84.5%; Bhutan/Myanmar: 5.5%; Iran: 4.5%; Sub-Saharan Africa: 4.5% | Australia | Adults:  F = 71.8%; M = 28.2%; Children: F = 44.4%; M = 55.6% | Women: *M* = 39.8 (*SD* = 8.4); Men: *M* = 44.9 (*SD* = 9.7); Girls: *M* = 11.9 (*SD* = 3.3); Boys: *M* = 12.0% (*SD* = 3.8) | PGD (4 items; Shear et al., 2011);  PTSD (PTSD-8); Trauma exposure (HTQ); Post-migration stressors (self-developed); Parenting style (Zubrick et al., 2014); Children’s psychological problems (SDQ) | ICD-11 | - Prevalence PGD (ICD-11): 36.8% (conditional) - Comorbidities: caregivers’ grief was directly associated with children’s emotional difficulties - Predictors: female gender; high age |
| Bryant et al. (2021a) | Cross-sectional (screening for an intervention study); Face-to-face-interview | *N* = 955  Treatment-seeking sample | Syria | Jordan | 67.3% female | Not reported | PGD (5-item interview consistent with ICD-11);  Psychological distress (K10); disability (WHODAS  2.0) | ICD-11 | - 59.1% reported bereavement - Prevalence PGD (ICD-11): 8.9%; (conditional prevalence 15.1%) - Comorbidities: more likely to have a serious mental disorder - No associations with marital status, educational level, cause of death, relationship to the deceased |
| Comtesse & Rosner (2019) | Cross-sectional study; Face-to-face-interview | *N* = 99  Convenience sample recruited from initial reception  centre and decentralized collective accommodations | Arabic: 45.4%; Kurdish: 32.3%; Afghan: 15.2%; Yazidi: 2.0%; Persian: 5.1% | Germany | 67.7% male | *M* = 30.1 (*SD* = 9.4) | PGD (TGI-SR);  PTSD (PCL-5); Depression (PHQ-9); Trauma exposure (LEC-5) | Prigerson et al., 2009; PCBD | - 92.0% reported bereavement - Prevalence PGD (Prigerson et al., 2009): 20.2% - Prevalence PCBD (DSM-5): 16.1% - Comorbidities: PTSD (44%); depression (11%); PTSD and depression (38%) - predictors: losses of nuclear family members; PTSD symptoms; temporary residence permit - No associations with age, gender, education, PTE, losses other than nuclear family members |
| Comtesse et al. (2021) | Cross-sectional study; Face-to-face-interview | *N* = 87  subsample from Comtesse & Rosner (2019) with confirmed or ambiguous loss | Confimed loss:  Arabic: 51.9%; Kurdish: 31.5%; Afghan: 12.9%; Persian: 3.7%  Ambiguous loss:  Arabic: 63.6%; Kurdish: 12.1%; Afghan: 18.2%; Persian: 6.1% | Germany | 67.8% male | *M =* 30.6 (range: 19–74) | PGD (TGI-SR);  PTSD (PCL-5); Depression (PHQ-9); Trauma exposure (LEC-5);  Perceived social support (FSSQ) | Prigerson et al., 2009 | - Prevalence PGD (Prigerson et al., 2009): 19.5%   - with confirmed loss: 14.8%   - with ambiguous loss: 27.2% - predictors: PTSD symptoms, (lack of) social support, ambiguous relative to confirmed loss, depressive symptoms - No associations with age, gender, number of losses, number of PTE, residence status, depressive symptoms |
| Craig et al. (2008) | Cross-sectional study; Self-report | *N* = 126  Random sample | Bosnia | USA | 56.0% female | *M* = 42.0 (*SD* = NR) | CG (ICG);  PTSD (PSDS); Depression (MHI); Anxiety (MHI); Trauma exposure (TLEQ); Well-being (MHI); General mental health (MHI) | CG | - 80.0% reported bereavement - Prevalence CG (scores ≥ 25 on the ICG): 54% - Correlations with mental health measures: PTSD; depression; anxiety; well-being; general mental health - Group differences: higher scores for female gender; age ≥ 55; no difference for years of education |
| Hinton et al. (2013a) | Cross-sectional study; Face-to-face-interview | *N* = 100  Treatment-seeking sample, bereaved | Cambodia | USA | 65.0% female | *M* = 54.2 (*SD* = 7.5) | PGD (PG-13);  PTSD (PCL); Losses (self-developed); Dream frequency (self-developed); Grief-related distress (self-developed) | Prigerson et al., 2009 | - Prevalence PGD (Prigerson et al., 2009): 8.0% (conditional) - Associations with mental health measures: 59.0% reported grief-related functional impairment - Correlates PGD: avoidance of reminders, rebirth concerns |
| Hinton et al. (2013b) |  |  |  |  |  |  |  |  | - Correlates PGD: frequency of dreams of the deceased |
| Kokou-Kpolou et al. (2017) | Cross-sectional study; Self-report | *N* = 74  (Migrants: *n* = 54; Refugees: *n* = 20)  Bereaved sample | Togo | Belgium, France | 62.2% male | *M* = 37.1 (*SD* = 11.03);  Migrants: *M* = 33.9 (*SD* = 10.0);  Refugees: *M* = 45.9 (*SD* = 8.7) | CG (ICG);  Death and ritual information (self-developed) | CG | - Prevalence CG (scores ≥ 31 on the ICG): 41.9% - Group differences & predictors: higher scores for refugee status, length of stay more than 10 years; loss of the mother; unexpected death; being eldest sibling; lower level of education; living in a couple; unemployment, no participation in bereavement rituals - No differences for gender, age, religious affiliation |
| Lacour et al. (2020) | Cross-sectional study; Self-report | *N* = 88  Clinical sample at different stages of treatment, bereaved (100% completed PGD measure) | Turkey: 59.1%;  Sri Lanka: 9.1%;  Iran: 6.8%; Iraq: 5.7%; Afghanistan: 3.4%;  Bosnia: 3.4%; Other: 12.5% | Switzerland | 80.7% male | *M* = 46.8 (*SD* = 9.6) | PGD (PG-13);  Trauma exposure (HTQ); Post-migration stressors (PMLDC); Emotion regulation (DERS); Self-efficacy (GSE) | Prigerson et al., 2009 | - Prevalence PGD (Prigerson et al., 2009): 23.9% (conditional) - Predictors: greater difficulties in emotion regulation; lower perceived self-efficacy - No associations with age, gender, length of stay, number of PTE, postmigration living difficulties |
| Nickerson et al. (2011) | Cross-sectional study; Face-to-face-interview | *N* = 315  Representative sample | Iraq (Mandaeans) | Australia | 52.5% female | *M* = 37.7 (*SD* = 14.7) | CG (ICG);  PTSD (HTQ); Depression (HSCL); Trauma exposure (HTQ); Mental health (SF-12) |  | - 78.4% reported bereavement - Pathways to CG in path analysis: loss; trauma, PTSD symptoms |
| Nickerson et al. (2014) | Cross-sectional study;  Face-to-face-interview | *N* = 248  Bereaved subsample from Nickerson et al. (2011) | Iraq (Mandaeans) | Australia | 52.0% female | *M* = 38.3 (*SD* = 14.5) | PGD (ICG);  PTSD (HTQ); Depression (HSCL); Trauma exposure (HTQ); Post-migration stressors (PMLDC); English language competency (ISLPR) |  | - Class membership in LCA: 17% PGD class, 16% PTSD/PGD class - Comorbidity PGD class: depression (75%) - Comorbidity PTSD/PGD class: depression (97.4%) - Predictors for PGD class membership: higher age; detention and abuse; adaption difficulties - Predictors PTSD/PGD class: female gender; detention and abuse; traumatic loss; loss of culture and support |
| Renner et al. (2021) | Cross-sectional (RCT baseline);  Face-to-face-interview | *N* = 47  treatment-seeking sample with ambiguous loss | Syria | Germany | 66.0% male | *M* = 34.79 years (*SD* = 12.32;  range = 19–64) | PGD (ICG);  PTSD (PDS-5) anxiety (GAD-7); depressive symptoms (PHQ-9), somatization (PHQ-15); boundary ambiguity (BAS) | CG | - Prevalence CG (scores ≥ 25 on the ICG): 42.6% (conditional) - Predictors: having lost a close family member, higher boundary ambiguity, age 30–39 vs. ≥40 - No association with closeness with missing person, age 30–39 vs. 18–29 |
| Silove et al. (2017) | Cross-sectional study; Face-to-face-interview | *N* = 230  Targeted sampling approach | West Papua | Papua New Guinea | 59.5% male | *M* = 37.0 (*SD* = 9.8) | PGD (R-MHAP restricted to ICD-11 symptoms)  PTSD (R-MHAP); CPTSD (R-MHAP); Depression (R-MHAP); Trauma exposure (self-developed); Post-migration stressors (ADAPT) |  | - Correlates: low correlations with PTSD (ICD-11) and cPTSD (ICD-11) |
| Tay et al. (2016) |  |  |  |  |  |  |  |  | - Predictors: conflict and loss related PTE, access to justice for human rights violations - No associations with sex, age, ADAPT domains bonds and networks, safety and security, roles and identities, existential meaning |
| Steil et al. (2019) | Cross-sectional study; Face-to-face-interview | *N* = 106  Representative sample | Afghanistan: 36.1%;  Syria: 32.5%; Iran: 10.8%; Iraq: 9.6%; Eritrea: 6.0%; Somalia: 2.4% | Germany | 100% female | *M* = 29.3 (*SD* = 8.8) | PGD (PG-13); Depression (HSCL); Anxiety (HSCL); Somatization (SCL-90); Trauma exposure (PDS, HTQ) | Prigerson et al., 2009 | - 84.9% of refugees reported bereavement - Prevalence PGD (Prigerson et al., 2009): 7.6%, 9.41% (conditional) - Correlations with mental health measures: moderate correlations with depression and anxiety; somatization - Predictors: family status; religion; somatization; number of experienced PTE - No associations with country of origin, level of education, having children, duration of displacement and application for asylum, age, number of witnessed trauma, depression and anxiety |
| Tay et al. (2019a) | Cross-sectional study; Face-to-face-interview | *N* = 486  Representative sample | West Papua | Papua New Guinea | 55.9% male | *M* = 35.8 (*SD* = 0.7) | PGD (R-MHAP);  PTSD (R-MHAP); Trauma exposure (self-developed); Postmigration living difficulties (HESPER) | ICD-11, PCBD | - 28.0% reported exposure to at least one type of traumatic loss - Prevalence PGD (ICD-11): 21% - Prevalence PCBD (DSM-5): 16% - Predictors: traumatic loss; longer duration of displacement - No associations with age, gender, employment, marital status |
| Tay et al. (2019b) |  |  |  |  |  |  |  |  | - Class membership in LCA: complicated bereavement class (11%); PTSD/complicated bereavement class (10%) - Predictor complicated bereavement class: erosion of interpersonal bonds/networks - Predictors PTSD/complicated bereavement class: exposure to traumatic loss events; post-migration stressors; erosion of interpersonal bonds/networks; disruptions to role and identity |

*Note*. Conditional prevalence rates pertain to the prevalence among bereaved individuals. CG = Complicated Grief; PCBD = Persistent Complex Bereavement Disorder; PGD = Prolonged Grief Disorder. ADAPT = ADAPT index of the psychosocial impacts of conflict and displacement; BAS = Boundary Ambiguity Scale; DERS = Difficulties in Emotion Regulation Scale; FSSQ = DUKE–UNC Functional Social Support Questionnaire; GSE = General Self-Efficacy Scale; HESPER = Humanitarian Emergency Settings Perceived Needs Scale; HSCL = Hopkins Symptom Checklist; HTQ = Harvard Trauma Questionnaire; ICG = Inventory of Complicated Grief; ISLPR = International Second Language Proficiency Rating Scale; K6 = Kessler Screening Scale for Psychological Distress; K10 = Kessler Psychological Distress Scale; LCA = latent class analysis; LEC-5 = Life Events Checklist; MHI = Mental Health Inventory; NR = not reported; PCL = Posttraumatic Stress Disorder Checklist; PCL-5 = PTSD Checklist for DSM-5; PDS = Posttraumatic Diagnostic Scale; PG-13 = Prolonged Grief-13; PHQ-9 = Patient Health Questionnaire Depression Module; PMLDC = Post-Migration Living Difficulties Checklist; PSDS = PTSD Screening and Diagnostic Scale; PTE = potentially traumatic event; PTSD-8 = Posttraumatic Stress Disorder-8 Items; R-MHAP = Refugee-Mental Health Assessment Package; SDQ = Strengths and Difficulties Questionnaire; SCL-90 = Somatization Subscale of the Symptom-Checklist-90; SF-12 = Medical Outcomes Study-Short Form; TGI-SR = Traumatic Grief Inventory Self-Report Version; TLEQ = Traumatic Life Events Questionnaire; WHODAS 2.0 = WHO Disability Assessment Schedule 2.0.
