# Supplementary material for: Prevalence, comorbidities, and factors associated with prolonged grief disorder, posttraumatic stress disorder and complex posttraumatic stress disorder in refugees: a systematic review
Source: Confl Health. 2024 Apr 16;18:32. doi: 10.1186/s13031-024-00586-5 (PMC11020800; doi:10.1186/s13031-024-00586-5)
Supplement: Supplementary file 4 — Supplementary Material 4. [file 13031_2024_586_MOESM4_ESM.docx]

Table 2.

*Study Characteristics and Main Findings for PTSD and cPTSD*

| Study | Study design & data collection | Sample size & population type | Country of origin | Host country | Gender | Age | Outcome variables (instrument) | Summary of main findings |
| --- | --- | --- | --- | --- | --- | --- | --- | --- |
| Barbieri et al. (2019) | Cross-sectional study; Face-to-face-interview | *N* = 120  Clinical sample receiving treatment for trauma-related mental health problems  At least one traumatic event | Nigeria: 26.7%; Ivory Coast: 15.8%; Gambia: 11.7%; Senegal: 9.2%; Ghana: 8.3%; Guinea Conakry, Sierra Leone: 5.0%; Democratic Republic of the Congo, Libya, Somalia: 2.5%; Cameroon, Egypt, Mali, Morocco: 1.7%; Benin, Congo-Brazaville, Guinea-Bissau, Mauritania, Sudan: 0.8% | Italy | 86.0% male | *M* = 25.1 (*SD* = 6.7) | PTSD (PCL-5); cPTSD (ITQ);  Trauma exposure (HTQ, PDS) | - Prevalence PTSD: 38.0% - Prevalence cPTSD: 30.0% - Predictors PTSD score: (fewer) months spent in Italy - LCA: cPTSD class (58.3%); PTSD class (41.7%) - No associations between class membership and legal status, gender, age, years of education, time in Italy, number of trauma types, employment |
| Barbieri et al. (2022) | Cross-sectional study; Face-to-face-interview | *N* = 126  Treatment-seeking sample  At least one traumatic event | Sudan (85.7%); Cameroun (5.5%); Central African Republic (4.0%); Mali (1.6%); Togo ( 1.6%); Burkina Faso (0.8%); Palestine (0.8%). | Niger | 75.4% male | *M* = 26.12 (*SD* = 6.88) | PTSD (ITQ); cPTSD (ITQ);  PTE (23 items, Nickerson et al., 2016)  Functional impairment (ITQ) | - Prevalence PTSD: 19.8 % - Prevalence cPTSD: 74.6% - LCA: cPTSD class (69.0%), PTSD class (31%) - cPTSD: more likely to live in Humanitarian Site, younger age at first trauma, increased levels of functional impairment - no association with age, gender, employment, number of trauma types, being with family |
| Frost et al. (2019) | Cross-sectional study; Face-to-face-interview | *N* = 308  Refugee subsample from a nationally representative survey | European: 23%; Asian: 22.6%; South American: 44.6%; African: 4.6%; North American: 4.3%; Other: 1% | USA | 51.3% male | *M* = 50.9 (*SD* = 17.7) | PTSD (ADADIS- IV); cPTSD (ADADIS- IV);  Trauma exposure (self-developed) | - 70.8% reported exposure to at least one traumatic event - prevalence PTSD: 20.9% - prevalence cPTSD (ICD-11): 4.9% - group differences: more females with cPTSD diagnosis - LCA: cPTSD class (13.3%); PTSD class (15.1%); PTSD low mood class (3.6%) - Predictors PTSD class: experience of being an unarmed civilian during war/revolution/military coup; serious accident - Predictors cPTSD class: physical assault; neglect; sexual assault; female gender; serious accident; increased amount of PTE - No association between class membership and natural disaster |
| Hecker et al. (2018) | Cross-sectional study; Face-to-face-interview | *N* = 94  Convenience sample recruited from counseling and information centers & language and integration  courses  At least one traumatic event | Syria: 29.8%; Afghanistan: 18.1%; Eritrea: 13.8%; Turkey: 6.4%; Iraq: 5.3%; North Africa: 5.3%; Sub-Saharan Africa: 4.3%; Other: 10.6%; Not specified: 6.4% | Switzerland | 85.1% male | *M* = 31.6 (*SD* = 10.1) | PTSD (ITQ); cPTSD (ITQ);  Depression (PHQ-9); Trauma exposure (HTQ); Post-migration stressors (PMLD); Social support (SPS) | - Prevalence PTSD: 32.9% - Prevalence cPTSD: 21.3% - Comorbidity PTSD: 25.8% at risk of moderate-to-severe depressive episodes - Comorbidity cPTSD: 80.0% at risk of moderate-to-severe depressive episodes - Predictor PTSD symptoms: trauma exposure - Predictors DSO: post-migration stressors; lack of social support - No associations between PTSD and lack of social support, gender, postmigration living difficulties - No associations between DSO and gender, trauma exposure |
| Heeke et al. (2020) | Cross-sectional study; Face-to-face-interview | *N* = 167  Clinical sample receiving outpatient treatment  traumatized | Syria: 42%; Afghanistan: 11%; Iraq: 10%; Turkey: 10% | Germany | 75.0% male | *M* = 34.2 (*SD* = 10.4) | PTSD (PCL-5);  Depression (HSCL); Anxiety (HSCL) | - Prevalence PTSD: 80.0% - Comorbidities PTSD: anxiety (97.0%); depression (99.0%) |
| Hyland et al. (2018) | Cross-sectional study; Face-to-face-interview | *N* = 110  Treatment-seeking sample  At least one traumatic event | Syria | Libanon | 80.2% female | *M* = 33.0 (*SD* = 8.9) | PTSD (ITQ); cPTSD (ITQ);  Trauma exposure (TLEQ); Functional Impairment (ITQ) | - Prevalence PTSD: 25.2% - Prevalence cPTSD: 36.1% - No gender differences - LCA: cPTSD class (64.5%); PTSD class (21.8%) - Predictors PTSD & cPTSD class: functional impairment - No associations between class membership and gender, unemployment status, trauma type, years of education, age, months spent in Lebanon, marital status, living status |
| Vallières et al. (2018) |  | *N* = 112 |  |  |  |  |  | - No associations between PTSD/cPTSD and gender, age |
| Jowett et al. (2021) | Cross-sectional study; Face-to-face-interview | *N* = 101,  ITQ data for *n* = 77  Treatment-seeking sample in specialist trauma service,  traumatized | 37 different nations | UK | 48.5% female | *M* = 34.56 (*SD* = 11.01) | PTSD (ITQ); cPTSD (ITQ);  PMLD (PMLDC); Well-being (CORE); | - prevalence PTSD: 14.3% - Prevalence cPTSD: 66.23% - Group differences PTSD & cPTSD: higher healthcare needs than refugees without theses diagnoses, postmigration living difficulties related to basic survival, integration, housing - Group differences cPTSD: greater levels of distress, higher relationship-related needs - No group differences for age, gender, being a victim of trafficking, type of trauma |
| Liddell et al. (2019) | Cross-sectional study; Face-to-face-interview | *N* = 112  Recruited for larger neuroimaging research project  51% referred from torture and trauma treatment service | Iran: 34.8%; Iraq: 20.5%; Afghanistan: 6.3%;  Sri Lanka: 6.3%; Other: 32.1% | Australia | 66.1% male | *M* = 37.7 (*SD* = 11.5) | PTSD (PSS-I); cPTSD (ITQ);  Trauma exposure (HTQ) | - LCA: affective dysregulation class (31.9%); cPTSD class (29.5%); PTSD-only class (23.5%); - Predictors PTSD class: higher trauma load; female gender, insecure visa status - Predictors cPTSD class: higher trauma load; female gender; insecure visa status - No associations between group membership and experience of torture above trauma load |
| Nickerson et al. (2016) | Cross-sectional study; Self-report | *N* = 134  Clinical sample receiving treatment for trauma-related mental health problems | Turkey: 53%; Iran: 12%;  Sri Lanka: 8%;  Bosnia: 5%; Iraq: 5%; Afghanistan: 4%;  Other: 13% | Switzerland | 78.4% male | *M* = 42.4 (*SD* = 9.8) | PTSD (PDS); cPTSD (PDS, DERS, HSCL, ECR);  Trauma exposure (HTQ, PDS) | - Prevalence PTSD: 19.7% - Prevalence cPTSD: 32.8% |
| Palic et al. (2016) | Cross-sectional study; Self-report | *N* = 116 refugees (N = 820 analyzed in total)  Treatment-seeking sample | Bosnia | Denmark | 52.6% female | *M* = 46.5 (*SD* = 8.1) | PTSD (HTQ); cPTSD (SIDES-SR);  Functional impairment(self-developed); Adverse childhood experiences (self-developed) | - LCA: cPTSD class (30%); PTSD class (18%) - cPTSD class associated with highest work-related impairment - Higher (c)PTSD symptoms among refugees compared to other traumatized groups in this study - No specific association between PTSD/cPTSD classes and adverse childhood experiences in refugee sample |
| Schiess‑Jokanovic et al. (2021) | Cross-sectional (baseline assessment from an RCT), face-to-face interviews | *N* = 93  Treatment-seeking sample | Afghanistan | Austria | 54.8% male | *M =* 34.77 years (*SD* = 13.84) | PTSD (ITQ); cPTSD (ITQ);  PTE (HTQ); postmigration living difficulties (PMLDC) | - Cluster analysis: cPTSD cluster (55.6%) & PTSD cluster (44.44%) - Characteristics of cluster membership: cPTSD cluster associated with more problems with language acquisition & barriers, higher total number of childhood traumatic event types - No associations between group membership and adult trauma types, family concerns, residence insecurity, and Socio-economical living conditions & discrimination |
| Schiess‑Jokanovic et al. (2022) |  |  |  |  |  |  |  | - prevalence PTSD: 17% - prevalence cPTSD: 50% - Network analysis: discrimination & socio-economical life conditions, language acquisition & barriers, and residence insecurity were connected to cPTSD symptom clusters No association of family concerns in the network |
| Silove et al. (2018) | Cross-sectional study; Face-to-face-interview | *N* = 487  Representative sample | West Papua | Papua New Guinea | 55.9% male | *M* = 35.8 (*SD* = 0.65) | PTSD (R-MHAP); cPTSD (R-MHAP);  Common mental disorders (R-MHAP); Trauma exposure (self); Postmigration living difficulties (HESPER); Functional impairment(WDODAS); Childhood adversities (ACE-IQ) | - Prevalence PTSD: 0.4% - Prevalence cPTSD: 9.3% - Comorbidity cPTSD: two or more other common mental disorders (97.8%) - Group differences: higher/more childhood adversities, trauma exposure, post-migration stressors, functional impairment in cPTSD vs. no disorder or common mental disorder groups - Predictors of cPTSD symptoms: childhood trauma & PTE associated with persecution and displacement, postmigration living difficulties - No associations with demographic variables |
| Tay et al. (2018) |  | *N* = 486 |  |  |  |  |  | - Predictors cPTSD: trauma exposure; post-migration living difficulties; functional impairment |
| Silove et al. (2017) | Cross-sectional study; Face-to-face-interview | *N* = 230  Targeted sampling approach | West Papua | Papua New Guinea | 59.5% male | *M* = 37.0 (*SD* = 9.8) | PTSD (R-MHAP, restricted to ICD-11 symptoms); cPTSD (R-MHAP, restricted to ICD-11 symptoms);  PGD (R-MHAP); Depression (R-MHAP); Trauma exposure (self-developed); Perceived insecurity and injustice (ADAPT) | - Prevalence PTSD: 6.0% - Prevalence cPTSD: 3.0% - Correlations among symptom counts for PTSD symptoms: high for cPTSD symptoms; moderate for PGD and depression - Correlations among symptom counts for cPTSD symptoms: high for PTSD; low for PGD; moderate for depression |
| Tay et al. (2015) |  |  |  |  |  |  |  | - 56.0% of refugees reported exposure to at least one type of human rights trauma - Group differences: higher PTSD symptoms in older, homeland born refugees |
| Vang et al. (2020) | Cross-sectional study; Face-to-face-interview | *N* = 284  Treatment-seeking sample with trauma-related mental-health problems | 24 nationalities across 6 regions:  Middle East (*n* = 177);  South-Eastern Europe (*n* = 80);  Caucasus (*n* = 8);  Asia (*n* = 7); Africa (*n* = 7); Northern Europe (*n* = 2) | Denmark | 52.5% male | *M* = 40.9 (*SD* = 9.8) | PTSD (ITQ); cPTSD (ITQ);  Trauma exposure (ITQ) | - Prevalence PTSD: 25.0%; - Prevalence cPTSD: 66.9% - LCA: cPTSD class (87%); PTSD class (13%) - No associations between class membership and age, gender, foreign citizenship, marital status, occupational status, need for interpreter, time since trauma, war trauma as index trauma, region of origin |
| Vang et al. (2021) | Cross-sectional study | *N* = 385 refugees (*N* = 1197 analyzed in total)  Treatment-seeking sample  Patients or patients’ relatives suffering from PTSD symptoms | Syria: 47.3% | Denmark | 51.9% male | *M* = 43.48, (*SD* = 10.02, range = 14–71) | PTSD (ITQ); cPTSD (ITQ);  Traumatic exposure (self-developed); anxiety & depression (HCSL) | - Prevalence PTSD: 23.3% - Prevalence cPTSD: 68.1% - Predictors for PTSD: female gender, higher age - Correlates with mental health measures: high associations between DSO and depression and PTSD and anxiety; low associations between PTSD and depression - No associations between PTSD and cumulative trauma, no associations between DSO and sex, age, cumulative trauma |

*Note*. DSO = disturbances in self-regulation; PTSD = posttraumatic stress disorder; cPTSD = complex posttraumatic stress disorder. ACE-IQ = Adverse Childhood Experiences International Questionnaire; ADAPT = ADAPT index of the psychosocial impacts of conflict and displacement; AUDADIS-IV = Alcohol Use Disorder and Associated Disabilities Face-to-face-interview Schedule-DSM-IV Version; CORE= Clinical Outcomes in Routine Evaluation; CSA = childhood sexual abuse; DERS = Difficulties in Emotion Regulation Scale; ECR = Experiences in Close Relationships Scale; ITQ = International Trauma Questionnaire; HESPER = Humanitarian Emergency Settings Perceived Needs Scale; HSCL = Hopkins Symptom Checklist; HTQ = Harvard Trauma Questionnaire; LCA = latent class analysis; LEC-5 = Life Events Checklist; NR = not reported; PCL-5 = PTSD Checklist for DSM-5; PDS = Posttraumatic Diagnostic Scale; PHQ-9 = Patient Health Questionnaire Depression Module; PMLDC = Post-Migration Living Difficulties Checklist; PSS-I = PTSD Symptom Scale-Face-to-face-interview Version; PTE = potentially traumatic event; R-MHAP = Refugee-Mental Health Assessment Package; SIDES-SR = Structured Interview of Disorders of Extreme Stress-Self Report; SPS = Social Provision Scale; MHPs = mental health professionals; WHODAS = WHO Disability Assessment Schedule.
